# Bioactivated Glucoraphanin Improves Cell Survival, Upregulating Phospho-AKT, and Modulates Genes Involved in DNA Repair in an In Vitro Alzheimer’s Disease Model: A Network-Transcriptomic Analysis

**DOI:** 10.3390/nu16234202

**Published:** 2024-12-05

**Authors:** Aurelio Minuti, Emanuela Mazzon, Renato Iori, Luigi Chiricosta, Osvaldo Artimagnella

**Affiliations:** 1 IRCCS Centro Neurolesi “Bonino-Pulejo”, Via Provinciale Palermo, Contrada Casazza, 98124 Messina, Italy; 2 Department of Innovative Technologies in Medicine & Dentistry, University “G. d’Annunzio” Chieti-Pescara, Via dei Vestini, 31, 66100 Chieti, Italy; emanuela.mazzon@unich.it; 3 Department of Food Quality and Nutrition, Research and Innovation Centre, Fondazione Edmund Mach (FEM), Via E. Mach 1, 38098 San Michele all’Adige, Italy

**Keywords:** Alzheimer’s disease, transcriptomic analysis, sulforaphane, glucoraphanin, isothiocyanate, DNA repair, cell survival

## Abstract

Background/Objectives: Alzheimer’s disease (AD) is one of the most common neurodegenerative diseases, for which a definitive cure is still missing. Recently, natural compounds have been investigated for their possible neuroprotective role, including the bioactivated product of glucoraphanin (GRA), the sulforaphane (SFN), which is highly rich in cruciferous vegetables. It is known that SFN alleviates neuronal dysfunction, apoptosis, and oxidative stress in the brain. In the light of this evidence, the aim of this study was to investigate the molecular effects of SFN pre-treatment in differentiated SH-SY5Y neurons exposed to β-amyloid (Aβ). Methods: To this end, we first evaluated first cell viability via the Thiazolyl Blue Tetrazolium Bromide (MTT) assay, and then we analyzed the transcriptomic profiles by next-generation sequencing (NGS). Finally, we used a network analysis in order to understand which biological processes are affected, validating them by Western blot assay. Results: SFN pre-treatment counteracted Aβ-induced loss of cell viability. The network-transcriptomic analysis revealed that SFN upregulates genes associated with DNA repair, such as *ABRAXAS1*, *BRCA1*, *BRCA2*, *CDKN1A*, *FANCA*, *FANCD2*, *FANCE*, *NBN*, and *XPC.* Finally, SFN also increased the phosphorylation of AKT, which is associated with DNA repair and cell survival. Conclusions: These data suggest that SFN is a natural compound that could be suitable in the prevention of AD, thanks to its neuroprotective role in increasing cell survival, potentially restoring DNA damage induced by Aβ exposure.

## 1. Introduction

Alzheimer’s disease (AD) is a progressive neurodegenerative disorder that slowly affects the brain, inducing pathological changes in the hippocampus, the center of memory and learning. AD is the most common form of dementia, and generally, people affected have prominent amnestic cognitive deficits [1]. Cognitive damages in AD patients can be different; indeed, they can be classified as presymptomatic or preclinical, mild, and severe [2]. The prevalence of AD is increasing worldwide. More than 55 million people suffer from AD or other dementias, with around 5 million new cases occurring every year [3].

To date, there are no cures for AD; only medications for moderating symptoms and clinical decline are available, but this global scenario demands new treatment strategies to prevent the onset and progression of AD. The cholinesterase inhibitors galantamine, rivastigmine, and donepezil; the N-methyl-D-aspartate (NMDA) receptor antagonist memantine; a combination of donepezil and memantine; and monoclonal antibodies targeting Aβ lecanemab and aducanumab are among the drugs approved by the US Food and Drug Administration (FDA) for the treatment of AD [4,5,6,7,8].

AD is molecularly characterized by β-amyloid (Aβ)-containing extracellular plaques and tau-containing intracellular fibrillary tangles. Altogether, AD leads to the accumulation of mis/unfolded proteins, oxidative stress, cell cycle dysregulation, DNA damage, and cell death, and particularly the loss of cholinergic neurons in the basal frontal lobe [9,10]. Double-strand breaks have been reported to accumulate in AD brains [11]. They occur at the promoter levels of several early response genes that are involved in memory and learning [12,13]. Senile plaques begin when the amyloid-β protein precursor (AβPP) is cleaved by α- and γ-secretases, resulting in Aβ aggregation and deposition in the brain [14]. Neurofibrillary tangles consist of hyperphosphorylated tau protein, which is associated with microtubules. Tau protein stabilizes microtubules when they are not phosphorylated [15].

An increasing number of studies have reported that many natural products activate the PI3K/AKT signaling pathway, which is fundamental in several processes such as neurogenesis, cell survival, neuronal proliferation, neurogenesis, and synaptic plasticity. The activation of PI3K/AKT pathway led to the protection of hippocampal, cortical, and dopaminergic neurons, and inhibited the activation of microglia, thus having a role in the prevention and treatment of neurodegenerative diseases such as AD [16].

**Figure 1:**
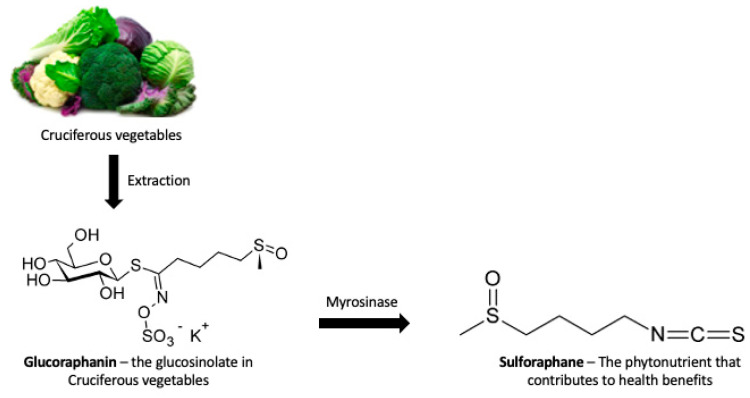
Chemical structures of glucoraphanin (GRA) and sulforaphane (SFN), along with a graphical representation of SFN’s biochemistry. Glucoraphanin is converted into sulforaphane through the action of the enzyme myrosinase. The chemical structures of GRA and SFN were obtained using PubChem compound records (accessed on 28 November 2024). Information about the molecule’s properties is available at the following links: https://pubchem.ncbi.nlm.nih.gov/compound/9548634, https://pubchem.ncbi.nlm.nih.gov/compound/Sulforaphane (both accessed on 28 November 2024).

In this regard, research regarding bioactive compounds is receiving more attention due to their multiple health benefits. Fruits, whole grains, and vegetables are good sources of bioactive chemicals [17]. Sulforaphane (1-isothiocyanato-4-methylsulfinylbutane, SFN) is one of these compounds, highly rich in cruciferous vegetables. It is an isothiocyanate, a bioactivated product of glucoraphanin (GRA), and belongs to the family of glucosinolates (GSLs). GSLs are made up of a sulfur-bonded β-D-glucopyranose residue, a hydroxylamine sulfate ester, and a variable aglycon side chain that is derived from an α-amino acid (R-group). The R-group classifies GSLs as aliphatic (from leucine, alanine, isoleucine, valine, or methionine) and aromatic (from tryptophan, tyrosine, or phenylalanine) [18]. GSLs are biologically inactive; through enzymatic hydrolysis by a glycoprotein named myrosinase (Myr), they undergo a modification of their chemical structure, producing several biologically active substances, such as thiocyanates, isothiocyanates, indoles, and so on (Figure 1) [19]. Sulforaphane is widely studied because it is a key regulator of cellular defenses, increasing or downregulating the expression of fundamental transcription factors [20]. Moreover, it has been shown that SFN attenuates inflammation, cell death, and oxidative stress in vivo models [21]. For instance, studies have shown that SFN exerts its neuroprotective effect also by stimulating the PI3K/AKT signaling pathway, as it did in primary cortical neurons and in transgenic AD mice [22]. Furthermore, we recently reported that SFN 1-5µM inhibited necroptosis and oxidative stress in an in vitro study [23]. Thanks to its properties, SFN could be considered a good candidate to prevent or ameliorate neurodegenerative diseases such as AD.

However, despite the many reports of SFN effects in vivo, little knowledge is available on the potential molecular mechanisms of its effects and on in vitro studies. In the light of this evidence, in this study, we evaluated the molecular effects of SFN pre-treatment in differentiated SH-SY5Y neurons exposed to β-amyloid (Aβ), a well-known in vitro model of AD. To this end, we took advantage of a network-transcriptomic analysis in order to understand which biological processes, and thus which genes, are modulated by SFN.

## 2. Materials and Methods

### 2.1. GRA Extraction, Purification, and Activation in SFN

The GRA isolation process was carried out in accordance with the guidelines established in CRA-CIN (Bologna, Italy) [24]. In detail, Tuscan black kale seeds were furnished by Suba Seeds (Longiano, Italy). Seeds were first crushed into a fine powder and then dehydrated in hexane. To inactivate the Myr enzyme, the solvent was removed and the solute was boiled with 70% ethanol to avoid GSL hydrolysis. Centrifugation was used to separate off the solid residue. Thus, GRA was isolated using one-step anion exchange chromatography and purified via gel filtration utilizing an XK 26/100 column filled with Sephadex G10 chromatography media (GE Healthcare, Chicago, IL, USA) in an AKTA-FPLC system (GE Healthcare).

Then, HPLC analysis was used to identify the fraction that contained pure SFN. According to the ISO 9167-1 method [25], GRA is pure when it is 99% (peak purity HPLC) and >95% weight basis (hydrated salt with 1-2 equivalents of water). The molar extinction coefficient value of 6634 L/(mol·cm) at 225 nm was determined using a Varian Cary 300 Bio UV/vis (Varian, Palo Alto, CA, USA) spectrophotometer. Later, the chosen fractions were combined and lyophilized [26]. Myr was isolated from the seeds of *Sinapis alba* [27]. The enzyme Myr has an activity of 35 U/mL and was stored at 4 °C in a sterile saline solution. A Myr unit is defined as the quantity of enzyme necessary to hydrolyze 1 μmol of sinigrin per minute at 37 °C and pH 6.5 [28]. GRA powder was dissolved in phosphate-buffered saline (PBS) 1× (Sigma-Aldrich, Saint Louis, MO, USA) (1 mg/mL). To bioactivate the GSL, we incubated GRA with 0.64 U of Myr at 37 °C for 1 h [29].

### 2.2. Cell Culture and Differentiation

The human neuroblastoma cell line SH-SY5Y was purchased from the American Type Culture Collection (ATCC) (Manassas, VA, USA). Cells were plated onto 96-well plates (Corning Incorporated, Corning, NY, USA) at a density of 60,000 cells/well or onto 6-well plates (ThermoFisher Scientific, Rochester, NY, USA) at a density of 1,500,000 cells/well in maintenance medium composed of DMEM/F-12 Ham (Sigma-Aldrich, Saint Louis, MO, USA) supplemented with 10% fetal bovine serum (FBS) (Sigma-Aldrich, Saint Louis, MO, USA), 1% penicillin/streptomycin (Sigma-Aldrich, Saint Louis, MO, USA), and 1% L-glutamine (Sigma-Aldrich, Saint Louis, MO, USA) at 37 °C in a humidified atmosphere of 5% CO_2_ and 95% air. The following day, SH-SY5Y cells were incubated for 5 days with 10 µM of retinoic acid (RA) (Sigma-Aldrich, Saint Louis, MO, USA) in order to induce differentiation.

### 2.3. Cell Treatment with β-Amyloid and SFN

After 5 days of RA-induced differentiation, SH-SY5Y cells were treated for 24 h with SFN at different concentrations (1 μM, 2.5 μM, and 5 μM). Differentiated SH-SY5Y were pre-treated with GRA along with homogeneous Myr instead of utilizing SFN directly because the chemical is poorly soluble and unstable in water [21]. SFN, obtained by bio-activation of GRA and Myr, was diluted in phosphate-buffered saline (PBS) 1× (Sigma-Aldrich, Saint Louis, MO, USA). Aβ (Sigma-Aldrich, Saint Louis, MO, USA) was dissolved in DMSO, diluted in PBS 1×, and finally aggregated at 37 °C for 24 h. It has been shown that Aβ incubation at 37 °C for 24 h induces the formation of aggregates [30]. At the end of the pre-treatment with SFN 5 μM, cells were treated with 10 µM of Aβ for 24 h. The concentration selected of Aβ was determined based on previous studies proving its capacity to induce cytotoxicity in SH-SY5Y cells [31,32,33]. Control cells were simply treated with PBS-diluted DMSO in the maintenance medium. The final DMSO concentration in cell cultures was <0.1%.

### 2.4. MTT Assay

SH-SY5Y cell cultures were cultured and treated with SFN and β-amyloid in 96-well plates as described above. At the end of treatment, cell viability was assayed with Thiazolyl Blue Tetrazolium Bromide (MTT) (Sigma-Aldrich, Saint Louis, MO, USA). In summary, cells were incubated in maintenance medium containing 0.5 mg/mL MTT for 4 h at 37 °C. The formed formazan crystals were dissolved in acidic (0.1 N HCl) isopropanol for 1 h at 37 °C. The absorbance was quantified by spectrophotometric measurement at 570 nm using the BioTek Synergy H1 microplate reader (Agilent, Santa Clara, CA, USA). The background was measured at 630 nm.

### 2.5. Extraction of RNA and cDNA Library Preparation

SH-SY5Y cells were cultured and treated in 6-well plates as above described. Subsequently, cells were harvested using 0.25% trypsin–ethylenediaminetetraacetic acid (EDTA) solution (#T4049, Sigma-Aldrich, Saint Louis, MO, USA) and centrifugated at 300× *g* for 5 min to obtain the cell pellet. Later, the pellet was processed for RNA extraction. Total RNA was extracted using the Maxwell^®^ RSC simplyRNA Cells Kit (#AS1390, Promega, Madison, WI, USA) with the Maxwell^®^ RSC instrument according to the instructions. Afterwards, library preparation (from 100 ng of total RNA) of two biological replicates was carried out using TruSeq^®^ RNA Exome (#20020189, #20020492, #20020183, #20020490, Illumina, San Diego, CA, USA) following the manufacturer’s instructions. The quality of the library was confirmed using the Tapestation 4150 instrument (Agilent, Santa Clara, CA, USA) with the D1000 screen tape (#5067-5582 and #5067-5583; Agilent, Santa Clara, CA, USA). A denaturation step using 0.2 N sodium hydroxide (NaOH) was performed, and then it was diluted until it reached a concentration of 1.42 pM. The NextSeq 500/550 Mid Output Reagent Kit v2.5 (150 cycles) (Illumina, San Diego, CA, USA) was employed in the sequencing with the Illumina instrument NextSeq^TM^ 550Dx (Illumina, San Diego, CA, USA). The paired-end mode was used for the run.

### 2.6. Transcriptomic Analysis

We verified the quality of the runs using FastQC (version 0.11.9, Babraham Institute, Cambridge, UK) and Trimmomatic (version 0.40-rc1, Usadel Lab, Aachen, Germany) was used to remove the adapters from the sequences [34]. The alignment and the counting of the reads were made using the STAR RNA-seq aligner (version 2.7.10a_alpha_220207, New York, NY, USA) [35] and HTSeq (version 0.13.5) [36], respectively, against the human reference genome (GRCh38). DESeq2 library version 1.36.0 was used in R (R Core Team) version 4.2.0 to obtain the fold change derived by the comparative analysis [37]. A post hoc correction of the *p*-value with threshold of 0.05 was adopted using the Benjamani–Hochberg procedure to remove the false-positive genes. The remaining genes were defined as Differentially Expressed Genes (DEGs). The database STRING was used to download protein–protein interaction networks [38]. ShinyGO was used to perform overrepresentation analysis [39].

### 2.7. Protein Extraction and Western Blot Analyses

SH-SY5Y was collected with trypsin–ethylenediaminetetraacetic acid (EDTA) at the end of the treatment, and RIPA (Thermo Scientific™, Waltham, MA, USA) was used to extract proteins in accordance with the manufacturer’s instructions. Protein concentration was evaluated using Bradford assay (Bio-Rad, Hercules, CA, USA). A total of 25 µg of proteins for each sample was separated on sodium dodecyl sulfate–polyacrylamide gel electrophoresis (SDS-PAGE) and transferred onto a PVDF transfer membrane (Immobilon-P PVDF, Merck Millipore division of Merck KGaA, Darmstadt, Germany). Subsequently, membranes were blocked for 1 h at room temperature with 5% skimmed milk in TBS and incubated with primary antibodies overnight at 4 °C. The primary antibodies used were the following: anti-p-Akt (#9271, 1:1000, Cell Signaling, Danvers, MA, USA), anti-Akt (#9272, 1:1000, Cell Signaling, Danvers, MA, USA), anti-cleaved caspase 3 (#9664, 1:1000, Cell Signaling, Danvers, MA, USA), and anti-GAPDH HRP conjugate (#3683, 1:1000, Cell Signaling, Danvers, MA, USA). The antibody mouse anti-rabbit IgG-HRP (sc-2357, 1:2000, Santa Cruz Biotechnology, Dallas, TX, USA) was employed as a secondary antibody to be incubated with the membranes for 1 h at room temperature. To visualize the expression of protein bands, an enhanced chemiluminescence system (Luminata Western HRP Substrates, Millipore Corporation, Billerica, MA, USA) was used, then protein bands were obtained and quantified with a ChemiDoc™ MP System (Bio-Rad Laboratories S.r.l., Hercules, CA, USA). Images were analyzed with ImageJ 1.54j software.

### 2.8. Statistical Analysis

Statistical analysis was performed using GraphPad Prism version 10.1 software (GraphPad Software, La Jolla, CA, USA). Multiple comparisons were carried out using one-way ANOVA tests and the Bonferroni post hoc tests. A *p*-value ≤ 0.05 was considered statistically significant. Data are expressed as mean ± Standard Error of the Mean (SEM).

## 3. Results

### 3.1. SFN Counteracts Cell Viability Loss Induced by Aβ Treatment

**Figure 2:**
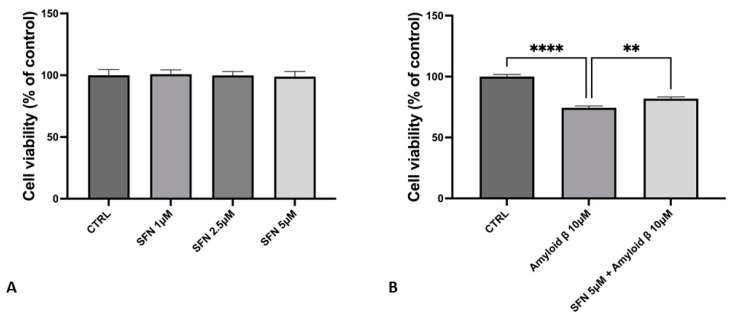
(**A**) Cell viability after SFN treatment. SFN at the concentrations tested did not affect cell viability. (**B**) Exposure with Aβ 10 µM decreased the cell viability of RA-SH-SY5Y-differentiated neurons. Interestingly, SFN at a concentration of 5 µM was able to increase cell viability. *N* = 6 independent biological replicates. Data are expressed as mean ± Standard Error of the Mean (SEM). ** *p* < 0.01; **** *p* < 0.0001. The complete primary data are reported in Table S1.

SH-SY5Y cells differentiated with RA were treated with different doses (1 µM, 2.5 µM, and 5 µM) of SFN for 24 h. By using the MTT assay, SFN exposure did not affect cell viability compared to the CTRL at all concentrations tested (Figure 2A). Then, to evaluate whether SFN was able to reduce Aβ toxicity, we pre-treated RA-differentiated SH-SY5Y neurons with the highest concentration used (SFN 5 µM), and with Aβ 10 µM for the next 24 h. MTT results highlighted that Aβ 10 µM treatment decreased the cell viability of RA-differentiated SH-SY5Y cells. Meanwhile, SFN 5 µM pre-treatment was able to restore cell viability (Figure 2B).

### 3.2. SFN Stimulates Genes Involved in DNA Repair Mechanisms

Based on MTT results, we performed a transcriptomic analysis of RA-differentiated SH-SY5Y cells treated with SFN 5 µM and Aβ 10 µM to assess the differentially expressed genes (DEGs). The comparative analysis was made on the Aβ sample against the SFN 5 µM + Aβ sample (Aβ vs. SFN 5 µM + Aβ). Aβ vs. SFN 5 µM + Aβ resulted in 1129 DEGs, among which 534 were downregulated and 595 were upregulated; they are showed in Table S2.

Thus, we constructed a network of Aβ vs. SFN 5 µM + Aβ DEGs, taking advantage of the protein–protein interaction database STRING (accessed on 14 October 2024). The analysis of the 1129 nodes showed 610 edges with an average node degree of 1.18 and an average local clustering coefficient of 0.253. We used the highest confidence score (0.900) and stringently removed interactions coming from text-mining, neighborhood, gene fusion, and co-occurrence sources.

The obtained network was then elaborated in R. Specifically, we selected and inspected in detail the subnetwork with the highest diameter counting 107 nodes, as shown in Figure S1. In Table S3, the details of the node interactions made by STRING are stored. The first main observation of this network was related to a cluster of mainly downregulated DEGs which were linked to ribosomal protein. The presence of ribosomal proteins seems to be associated with the activation of translational machinery triggered by the presence of SFN 5 µM. Nevertheless, the cluster hindered the discovery of additionally mechanisms. For this reason, we used the HUGO Gene Nomenclature Committee website (accessed on 14 October 2024) to retrieve the genes associated with ribosomes. We identified for ribosomal proteins (group id 1054) the most general categorization of these proteins. Thus, we removed from the previous network the 21 nodes included in group 1054 and 86 DEGs were kept.

**Figure 3:**
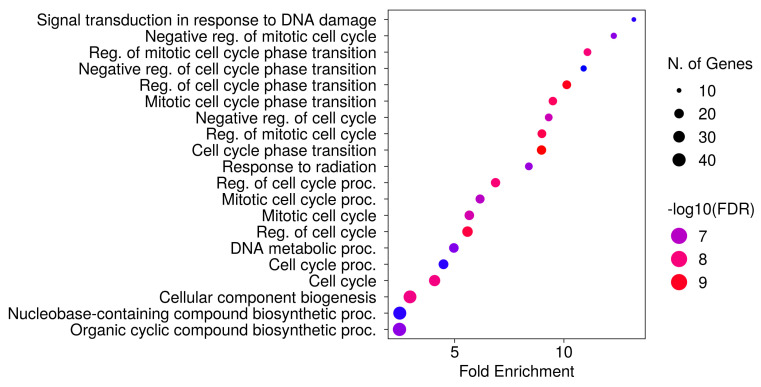
Cluster of over-represented biological terms obtained by ShinyGO. The terms are sorted by fold enrichment after FDR. The color palette shows the log FDR. The size of the bubble represents the number of DEGs in the cluster.

We searched for the main biological process linked to the 86 remaining DEGs using ShinyGO (accessed on 16 October 2024). ShinyGO clusterization of biological process terms over-represented 487 terms with a false discovery rate (FDR) under the significant threshold of 0.05. Interestingly, as shown in Figure 3, the most over-represented term by fold enrichment and FDR sorting was “Signal transduction in response to DNA damage” (GO:0042770).

Additionally, many clusters of over-represented biological terms with lower fold enrichment are correlated with the cell cycle and its regulation. In particular, the most specific children are related to the mitotic cell cycle, following negative regulation.

SFN regulates important genes involved in DNA repair, protecting neurons from DNA damage induced by Aβ. DEGs included in this ontology term are *NBN*, *FANCD2*, *FANCA*, *FANCE*, *BRCA1*, *BRCA2*, *CDKN1A*, *XPC*, *KDM1A*, and *ABRAXAS1* and their deregulation levels are given in Table 1.

**Figure 4:**
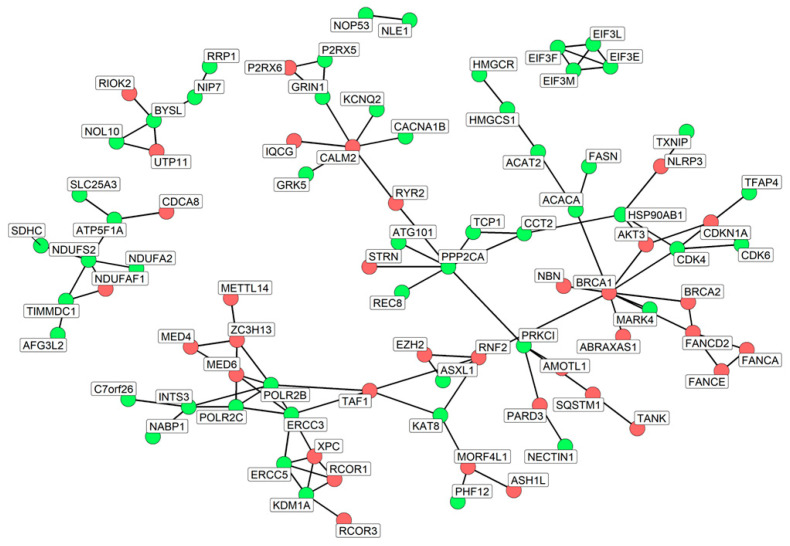
Final subnetworks of DEGs included in the network with the highest diameter excluding ribosomal proteins. The color of the nodes represents the deregulation of DEGs; green nodes are downregulated DEGs while red nodes are upregulated.

Additionally, for the 86 nodes, we also computed the network that is depicted in Figure 4.

**Figure 5:**
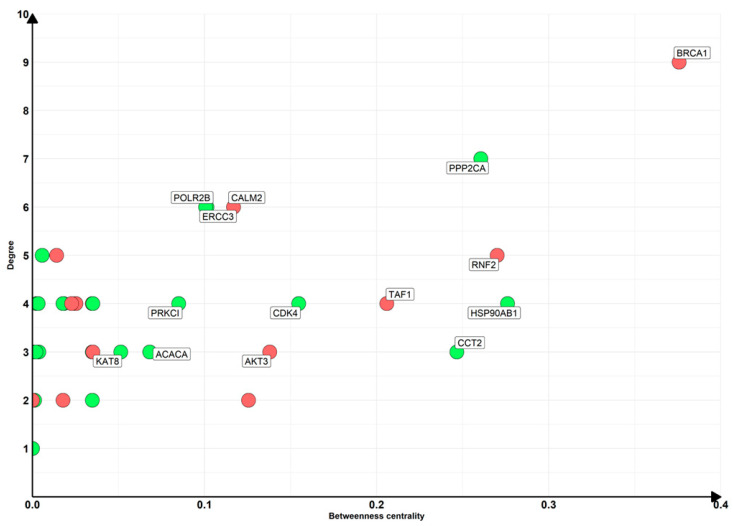
Degree and betweenness score values for DEGs included in the network with the highest diameter excluding ribosomal proteins. The color of the nodes represents the deregulation of DEGs so that green nodes are downregulated while red nodes are upregulated DEGs. Labels are shown only for nodes with betweenness higher than 0.5 and degree higher than 2 for higher readability.

Along with their biological roles, we inspected the network properties to find the most important nodes. In this sense, we focused our attention on the hub nodes, inspecting the values of degree and betweenness centrality, and the scores are given in Table S4. Interestingly, as highlighted in Figure 5, the node with the highest degree and simultaneously the highest betweenness centrality is *BRCA1*. Curiously, as previously illustrated in Figure 4, its interactors, *BRCA2*, *NBN*, *FANCD2*, and *ABRAXAS1*, are all upregulated and strictly interact with each other, forming a cluster.

**Figure 6:**
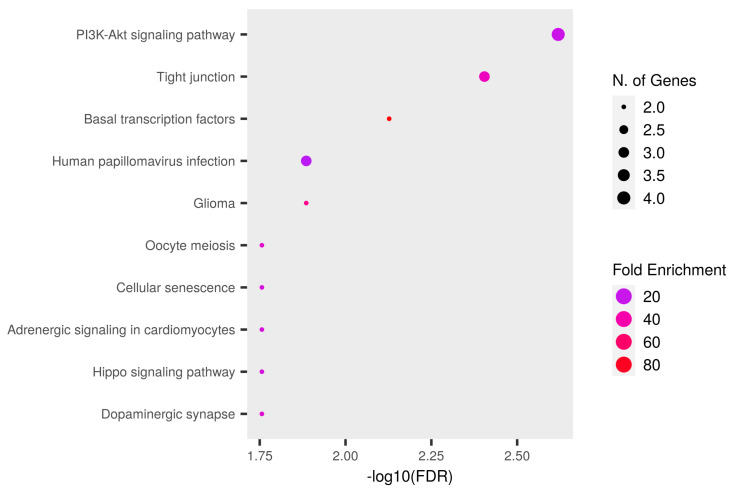
KEGG pathway terms obtained by ShinyGO. The terms are sorted by FDR. The color palette shows the fold enrichment. The size of the bubble represents the number of DEGs in the cluster.

Then, we performed over-representation analysis of nodes with the most extreme scores. In detail, we chose *BRCA1*, *PPP2CA*, *RNF2*, *HSPA90AB1*, *CCT2*, *TAF1*, *CDK4*, *AKT3*, *PRKCI*, *ACACA*, *KAT8*, *CALM2*, *ERCC2*, and *POLR2B* because they had betweenness higher than 0.5 and degree higher than 2. ShinyGO analysis of these nodes revealed after FDR sorting the most over-represented KEGG pathway, the “PI3K-AKT signaling pathway”, as shown in Figure 6. This pathway is known to be essential to molecular signaling in the central nervous system. Additionally, the overrepresentation of the tight junction pathway in neuronal cells suggests that the molecular processes and proteins involved in the formation and maintenance of tight junctions are more actively engaged [40]. Tight junctions also play a role in cell signaling and in modulating interactions between neuronal cells. Changes in their presence or function may be associated with processes such as neuronal maturation or the development of neural circuits. In this regard, even the enrichment of the “Dopaminergic synapse” KEGG pathway may suggest a role of SFN in improving synaptic plasticity.

Conversely, the KEGG pathways “Human Papillomavirus Infection”, “Glioma”, and “Basal Transcription factor” are not relevant to the context of this study.

Based on the number of genes, SFN may primarily stimulate the PI3K-AKT pathway, as accords with our data, as shown in Figure 6.

### 3.3. Western Blot Analyses Revealed Activation of AKT and Inhibition of Apoptosis

**Figure 7:**
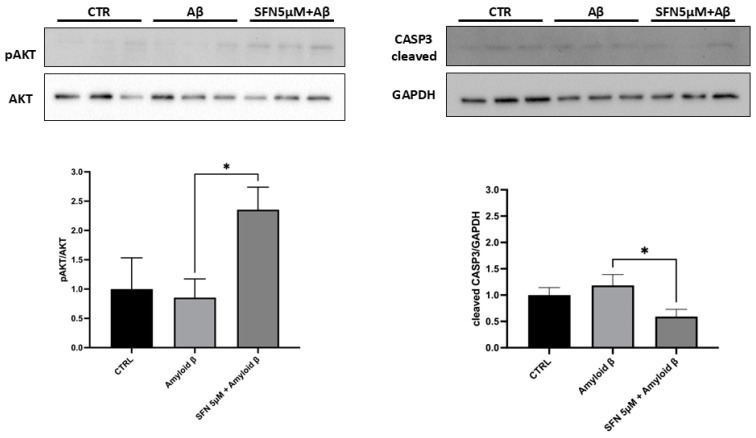
Western blot analysis for p-AKT and cleaved-CASP3. (**Left**) Aβ treatment induced a light decrease in p-AKT, while SFN increased p-AKT levels. (**Right**) In addition, SFN treatment reduced levels of cleaved-CASP3 compared to Aβ. *N* = 3 independent biological replicates. The results are indicated by mean ± Standard Error of the Mean (SEM). * *p* < 0.05. The complete primary data are reported in Table S1.

In order to validate that the PI3K-AKT pathway is stimulated by SFN and, thus, its effects on cell survival and apoptosis, we evaluated the protein levels of p-AKT and cleaved CASP3, respectively. Western blot analysis evidenced a strong increase in p-AKT and a strong decrease in cleaved CASP3 in Aβ vs. SFN5 + Aβ (Figure 7), confirming that SFN pre-treatment stimulated the PI3K-AKT pathway and inhibited apoptosis in the AD model.

Altogether, these results suggest that SFN pre-treatment may increase the cell survival of differentiated SH-SY5Y neurons exposed to Aβ, upregulating genes involved in DNA repair, phosphorylating AKT protein, and decreasing cleaved CASP3 levels.

## 4. Discussion

AD is a neurodegenerative disorder that affects millions of people worldwide and is still without an efficacious treatment. Aβ originates from amyloid precursor protein (APP) cleaved by β-secretase and γ-secretase. In AD, the activity of Aβ-degrading enzymes is reduced, leading to Aβ accumulation [41]. Aβ_1–42_ is more toxic and its accumulation has several neurotoxic effects, leading to mitochondrial dysfunction, synaptic damage, neuronal death, and extensive DNA damage [42]. In light of this evidence, preventive measures are needed to avoid neuronal dysfunction.

In this regard, bioactive compounds have been recently studied for their neuroprotective roles both in in vitro and in vivo models [43,44,45].

Concerning the prevention of neurodegenerative diseases, the isothiocyanate SFN is receiving more attention as a possible compound aimed at improving neurodegenerative disorders. SFN has the capacity to cross the blood–brain barrier, resulting in elevated bioavailability [46]. In the context of AD, SFN has the capacity to counteract Aβ accumulation, promoting memory and spatial learning in AD transgenic mice [47], indeed it was demonstrated that SFN downregulated proteins implicated in the amyloidogenic pathway [47]. Moreover, we recently reported that SFN, in the range of 1–5 µM, inhibits necroptosis and oxidative stress in RA-differentiated NSC-34 motoneurons [23].

In this study, we performed a transcriptomic analysis in order to characterize molecular mechanisms underlying neuroprotective functions exerted by SFN in an in vitro model of AD. For this reason, we used RA-differentiated SH-SY5Y cells exposed to extracellular Aβ 10 µM for 24 h. The SH-SY5Y cell line is widely used to produce an in vitro AD model because the cells therein can differentiate into cholinergic neurons [48,49].

Thus, the initial phase of our research was to evaluate the effects of SFN on cell viability by MTT assay. SFN did not affect the cell viability of RA-differentiated SH-SY5Y neurons at a concentration range of 1–5 µM (please refer to Figure 2). Importantly, we showed that SFN at a concentration of 5 µM was able to remedy the cell viability loss caused by Aβ. Based on these results, we performed transcriptomic analysis, using the 5 µM concentration. Subsequently, to evaluate biological processes of the DEGs found in the Aβ vs. SFN5µM + Aβ comparison, we analyzed protein–protein interactions using the STRING database. Interestingly, DEGs included in the biggest network were mainly involved in signal transduction in response to DNA damage. In particular, we found that key genes belonging to DNA cell repair were all upregulated, including *BRCA1*, *BRCA2*, *ABRAXAS1*, *NBN*, *FANCA*, *FANCD2*, *FANCE*, *XPC*, and *CDKN1A.*

The central nervous system is particularly sensitive to failure to repair DNA [50] and is linked to high levels of transcription activities and metabolism of neurons [51,52]. Base excision repair (BER) is the main DNA repair mechanism that addresses small base alterations caused by processes like alkylation, deamination, and oxidation. It is believed to be crucial for both the development and ongoing maintenance of the central nervous system [53]. Defects in BER have been observed in brain regions affected by AD, suggesting that BER dysfunction may be a common feature of AD [54]. Furthermore, BER deficiencies have been associated with replication errors that lead to double-strand breaks (DSBs) [55]. DSBs take place in the promoters of several early response genes, contributing to learning and memory deficits [56]. In transgenic mice of human APP mutant, an increasing number of DSBs were found [57]. DSBs are repaired by homologous recombination (HR) or non-homologous end-joining (NHEJ) [58]. DSBs activate DNA-PK (DNA-dependent protein kinase) and ATM (ataxia-telangiectasia mutated) proteins [59]. The MRE11-RAD50-NBN complex (MRN) acts as a mediator to recruit ATM to DSBs; then, ATM activates BRCA1 to induce end resection to repair DSBs by HR [58]. In detail, histone γH2AX phosphorylation at serine 139 mediated by the ATM kinase leads to a new binding site for the BRCA1 C-terminal domain of the Mediator of DNA damage Checkpoint protein 1 (MDC1) [60]. MDC1 positioning is fundamental to correctly recruit the AT kinase and the MRN complex [61]. Successively, MDC1 promotes ubiquitin ligases RNF168 and RNF8, favoring the loading of BRCA at the DSB sites [62]. NHEJ is the first option for DSBs repair, particularly in non-dividing cells such as neurons [58]. Indeed, DNA-PK can also be activated by DSBs via the KU70/Ku80 complex, then activating 53BP1, committing to NHEJ-mediated DSB repair [63]. The heterodimer Ku70-Ku80 binds to the DSB, and BRCA1 strengthens the heterodimer at the DSB sites [64].

Although *BRCA1* and *BRCA2* are well-known breast cancer susceptibility genes [65], ever more studies are correlating them with many other scenarios [66], due to their main role in DNA repair mechanisms (as described above).

*BRCA1* plays a role in maintaining genomic stability, acting also as a tumor suppressor. Indeed, individuals carrying loss-of-function mutations have a higher susceptibility to ovarian and breast cancer, increasing the persistence of DSBs [67]. However, several studies have shown the correlation between DNA damage and neurodegenerative disorders, such as AD, observing defects in DNA repair mechanisms [68]. In this regard, in an AD model, brain of mice with knockdown of *BRCA1* exhibited increased DSBs with learning and cognitive deficits [69]. Furthermore, in AD brains, it was found that the BRCA1 protein was mis-located into the cytoplasm complexed with tau, reducing nuclear protein levels. This suggests that BRCA1 is not functional concerning DSB repair [70]. In addition, downregulations in BRCA1 have been reported in hippocampal neurons of mild cognitive impairment (MCI) and AD brains, suggesting that *BRCA1* plays an essential role in AD [71]. Furthermore, studies have highlighted that the increase in DSBs is due to the overexpression of NMDA (*N*-methyl-D-aspartate) receptors that induce the degradation of *BRCA1* levels [57]. AD hyperactivates NMDA receptors, leading to a reduction in BRCA1 levels in mice, which induces an impaired neuronal DNA damage response [69]. This suggests that Aβ accumulation directly induces DNA damages. Interestingly, in our data, we found a downregulation of *GRIN1* (Table S2) gene expression in Aβ vs. Aβ + SFN5µM, which supports the upregulation of *BRCA1* and the beneficial role of SFN.

Mutations of *BRCA2* are also linked with breast cancer. In physiological conditions, *BRCA2* is involved in the maintenance of genome stability, acts as a tumor suppressor, and is also involved in DSB repair. Indeed, cells lacking *BRCA2* are not capable of repairing DSBs because of impaired HR and NHEJ mechanisms [67]. *BRCA2*, once activated by *BRCA1*, binds RAD51 and increases DNA repair by promoting the assemblage of RAD51 onto single-strand DNA (ssDNA) [72]. *BRCA2* is necessary to prevent R-loop-associated DNA damage, and consequently, genomic instability [72]. Subsequently, *BRCA2* interacts and activates components of the Fanconi anemia (FA) core complex [72].

We found that some of these genes were upregulated, in particular *FANCA*, *FANCD2*, and *FANCE*. *FANCA* is involved in DNA cross-link repair and maintains normal chromosome stability [73]. *FANCA* downregulation is correlated with enhanced instability at stalled replication forks; indeed, it is necessary to maintain the stability of chromosomal fragile sites [74]. *FANCD2* cooperates with *FANCA*, *FANCE*, and *BRCA1* to prevent loss and breaking of mis-segregating chromatin at the end of cell division, in particular after replication stress. *FANCD2* promotes FANCD1/BRCA2 loading onto impaired chromatin [75,76]. Studies support the claim that BRCA1, BRCA2, and FANCD2 promote the protection of nascent strands by the nucleases DNA2 and MRE11 loading RAD51 onto the fork [72].

Moreover, we found that the w*ABRAXAS1* gene was upregulated, which is an adapter of the BRCA1-A complex; the encoded protein leads to the recruitment of BRCA1. Interestingly, BRCA1-A physically links BRCA1 at DSBs via RAP80, MERIT40, BRCC36, and BRCC45 [77], prevents the excessive resection sequestering BRCA-1 to the flanks of DSBs [78], and ends the DNA damage response (DDR) via BRCC36 activity [79]. In detail, the ABRAXAS1/BRCA1-A complex counteracts break-induced replication (BIR) and inhibits DNA end resection by limiting K63-linked ubiquitin modification catalyzed by Ubc13/RNF168. When BRCA1-A is downregulated, increased K63-linked ubiquitin induces MUS81/SLX4 excess on chromatin, which determines single-ended DSBs, successively processed by the endonucleases CTIP, DNA2/BLM, and MRE11, creating an extended length of ssDNA. When *ABRAXAS1* is dysregulated, the excessive loading of MUS81 induces severe chromosome abnormalities and increases mitotic DNA synthesis, causing cell death [80]. Thus, *ABRAXAS1* is a crucial gene involved in DNA repair.

Although neurons are post-mitotic cells, cell cycle re-entry can occur in the case of DNA damage and oxidative stress [81]. This can induce apoptosis or senescence, depending on the amount of DNA damage and the degree of cell cycle progression [82]. Interestingly, progression from G_0_ to G_1_ is correlated with increased NHEJ; however, persistent DNA damage and improper DNA repair may lead to cellular senescence [83].

In our data, we found that SFN treatment upregulated the *CDKN1A* gene. This gene encodes a cyclin-dependent kinase inhibitor (p21), and it plays a pivotal role in controlling DNA-damage [84]. It is induced by *BRCA1* in response to DNA damage [85]. It has been shown that SFN is able to increase p21 protein expression, in accordance with our transcriptomic data [43]. SFN upregulates p21 in response to DNA damage [86]. *CDKN1A*, being an important CDK inhibitor, reduces the expression of cyclin D-CDK4 complex. Of note, we found that *CDK4* and *CDK6* were downregulated, which supports their downregulation as mediated by SFN. *CDK4* is a catalytic subunit important for cell cycle progression [87]. Similar to *CDK4*, *CDK6* mediates cell cycle progression [87].

SFN 5 μM treatment increased the expression of *XPC*, which is specifically induced by *BRCA1* [88]. This gene plays an important role in nucleotide excision repair, and it recognizes an extensive variety of DNA damaged such as mismatched bubbles, single-stranded loops, and single-stranded overhangs [89].

We also found that the gene *NBN* was upregulated; this gene is a member of the MRN complex and plays a key role in DSB repair, the maintenance of telomere integrity, and DNA recombination [90]. In the MRN complex, *NBN* acts as a protein–protein adapter, which activates and recruits RAD50 and MRE11 components to DNA damage sites [91]. Furthermore, the upregulation of the *NBN* complex increases p-AKT levels through interaction with the mTOR/Rictor/*SIN1* complex [92]. Interestingly, *NBN* activates the PI3K/Akt signaling pathway through interacting via its conserved C-terminal domain (aa 653-669) with the mTOR/Rictor complex [92,93]. The knockdown of *NBN* induced a downregulation of p-AKT levels, demonstrating the pivotal role of *NBN* in increasing p-AKT levels [92].

Interestingly, it was demonstrated that AKT signaling directly phosphorylates BRCA1 in different sites, such as on T509 and S694, thereby activating and regulating the nuclear localization of BRCA1, enabling BRCA1 to be able to intervene in DNA repair [94]. Several studies demonstrated the association between the PI3K/Akt signaling pathway and AD [95,96]. When AKT is not phosphorylated, it is responsible for several events related to AD, including Tau hyperphosphorylation [97]. Of note, p-AKT may increase cell survival, blocking apoptotic signals [98]. In mature neurons, AKT has a pivotal role in neuronal polarity, metabolic control, neurotransmission, synaptic plasticity, and stress response including DNA damage repair [99]. Once AKT is upregulated, neuron survival improves, so approaches aimed at activating AKT may be useful for improving neuronal survival and inhibiting AD pathology [100]. In addition, studies have shown that SFN exerts its neuroprotective effect by activating the PI3K/AKT signaling pathway. For example, it increases levels of TrkB and its signaling molecules p-CREB, p-CaMKII, p-ERK, and p-AKT in primary cortical neurons and in transgenic AD mice [22]. Moreover, SFN has been shown to activate the AKT pathway in different cell lines exposed to heavy metals [101,102,103], inducing the upregulation of several target genes linked with inflammation and apoptosis. Finally, an inhibitor of the PI3K/AKT signaling pathway (LY294002) blocked the beneficial effects of SFN in PC12 cells [104].

Remarkably, in validating our transcriptomic data, we obtained a significant increase in p-AKT and a decrease in cleaved CASP3 (please refer to Figure 7), suggesting that SFN stimulated cell survival and inhibited apoptosis in our AD model.

## 5. Conclusions

**Figure 8:**
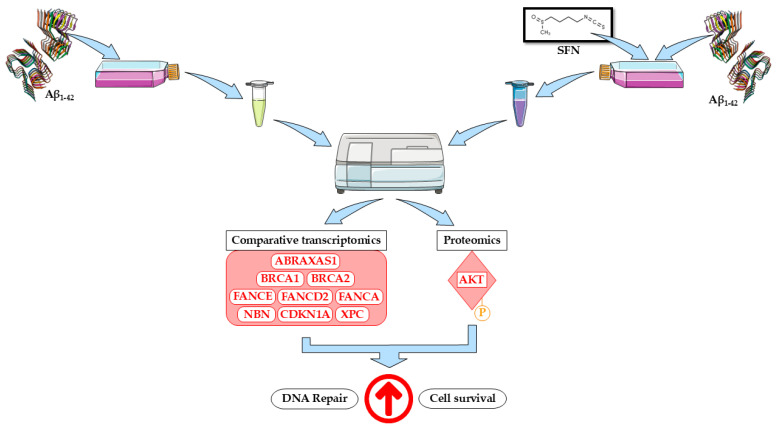
Schematic representation of putative molecular mechanism of how SFN could be involved in DNA repair process and cell survival in AD model. In red we indicated all genes/proteins upregulated. The arrow in red indicates the upregulation of DNA repair and cell survival process. β-amyloid structure (9CZP) was retrieved by PDB. Figure was drawn using vector image bank of Servier Medical Art by Servier (smart.servier.com) (accessed on 3 November 2024). Licensed under Creative Commons Attribution 3.0 Unported License (creativecommons.org/licenses/by/3.0/) (accessed on 3 November 2024).

This study elucidates a possible molecular mechanism whereby SFN stimulates the PI3K-AKT pathway via upregulation of p-AKT, resulting in downstream increases in *BRCA1* and other key genes that act to restore DNA damage induced by Aβ (Figure 8). Furthermore, SFN inhibits apoptosis by reducing the protein levels of cleaved CASP3. Further studies are needed to better elucidate what concentration of SFN is useful for counteracting the effects of Aβ accumulation.

This work’s strengths have to do with its transcriptomic analysis, which allowed the investigation of genes involved in different signaling pathways. Moreover, we applied a stringent filter to our data in order to help us identify the biological processes and molecular functions related to the exposure to SFN in RA-differentiated SH-SY5Y cells treated with Aβ and we evaluated protein levels of key genes involved in cell survival and apoptosis by Western blot.

In light of our data, we can hypothesize that SFN has a neuroprotective role and it could be a useful adjuvant to prevent AD.

## Figures and Tables

**Table 1:** DEGs included in biological term signal transduction in response to DNA damage.

Gene	Expression Aβ	Expression SFN 5 µM + Aβ	Fold Change	qValue
*ABRAXAS1*	257.87	311.56	0.27	2.91 × 10^−2^
*BRCA1*	7573.63	8576.82	0.18	1.50 × 10^−5^
*BRCA2*	6125.88	6979.65	0.19	1.76 × 10^−3^
*CDKN1A*	721.77	1039.85	0.53	2.32 × 10^−3^
*FANCA*	10,898.09	11,329.24	0.06	1.95 × 10^−3^
*FANCD2*	13,431.01	14,889.88	0.15	2.31 × 10^−2^
*FANCE*	1892.62	2051.59	0.12	9.40 × 10^−3^
*KDM1A*	10,353.58	9712.88	−0.09	3.92 × 10^−2^
*NBN*	1756.94	1911.43	0.12	9.29 × 10^−3^
*XPC*	5391.15	5912.85	0.13	1.50 × 10^−9^

## Data Availability

The data presented in this study are openly available in the NCBI Sequence Read Archive at BioProject, accession number PRJNA1179938.

## Supplementary Materials

The following supporting information can be downloaded at: https://www.mdpi.com/article/10.3390/nu16234202/s1, Figure S1: Picture of main STRING network of DEGs; Figures S2–S5: Uncropped Western blot images; Table S1: Primary data of MTT and Western blot assays; Table S2: DEGs obtained by transcriptomics analysis; Table S3: Protein–protein interactions of main STRING network of DEGs; Table S4: Scores related to hub nodes of simplified main network.
