# Supplementary figures and images for: Bioactivated Glucoraphanin Improves Cell Survival, Upregulating Phospho-AKT, and Modulates Genes Involved in DNA Repair in an In Vitro Alzheimer’s Disease Model: A Network-Transcriptomic Analysis
Source: Nutrients. 2024 Dec 5;16(23):4202. doi: 10.3390/nu16234202 (PMC11644231; doi:10.3390/nu16234202)

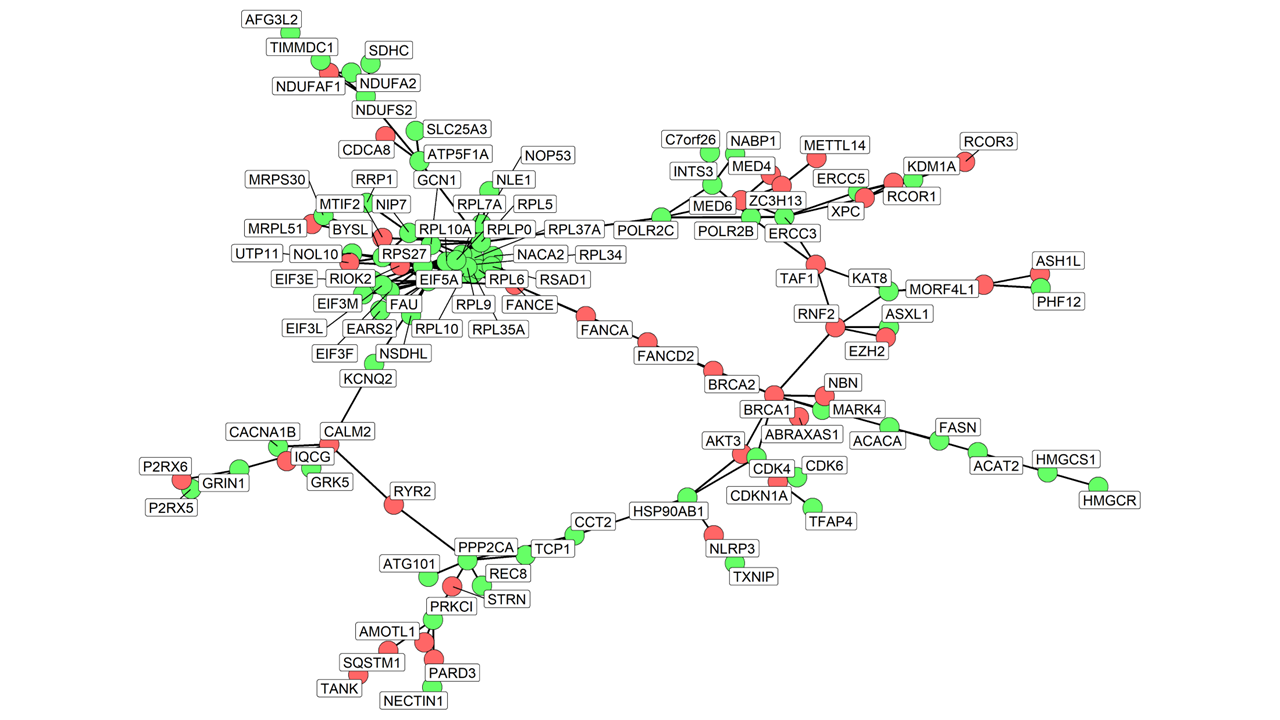

Supplement: Supplementary file 1 [file nutrients-16-04202-s001.zip › Figure S1.TIF]

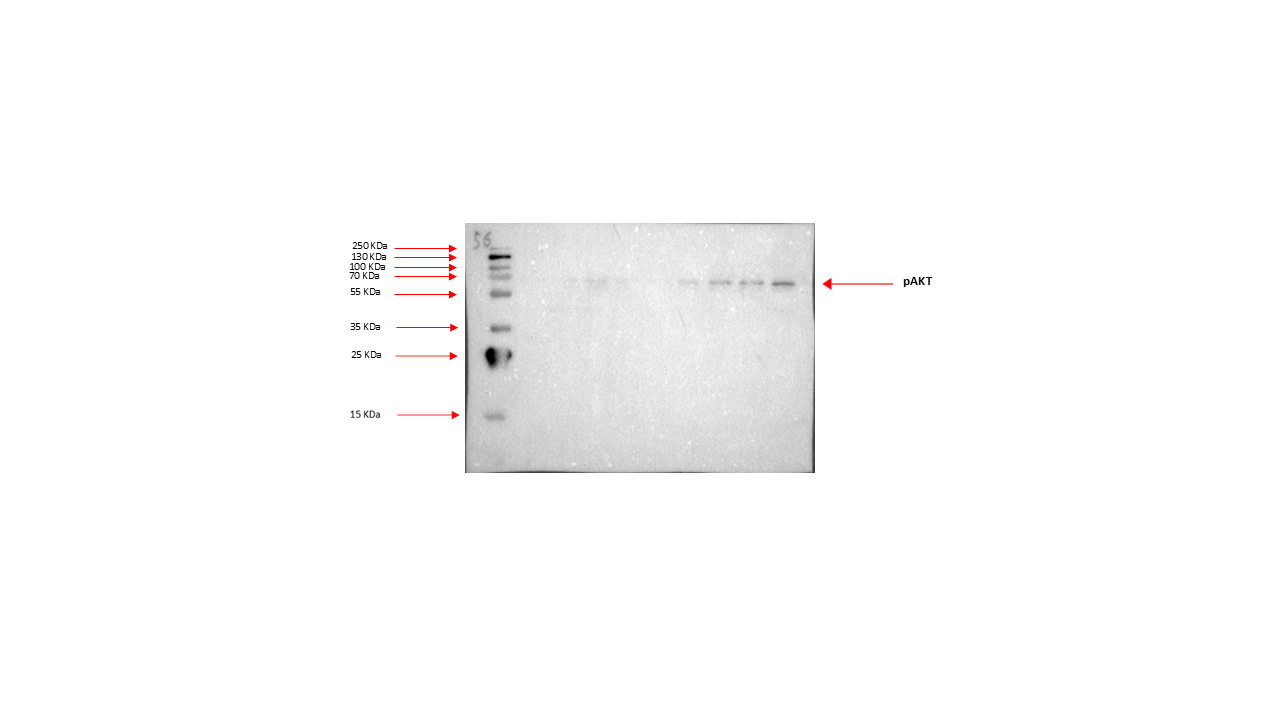

Supplement: Supplementary file 1 [file nutrients-16-04202-s001.zip › Figure S2.tif]

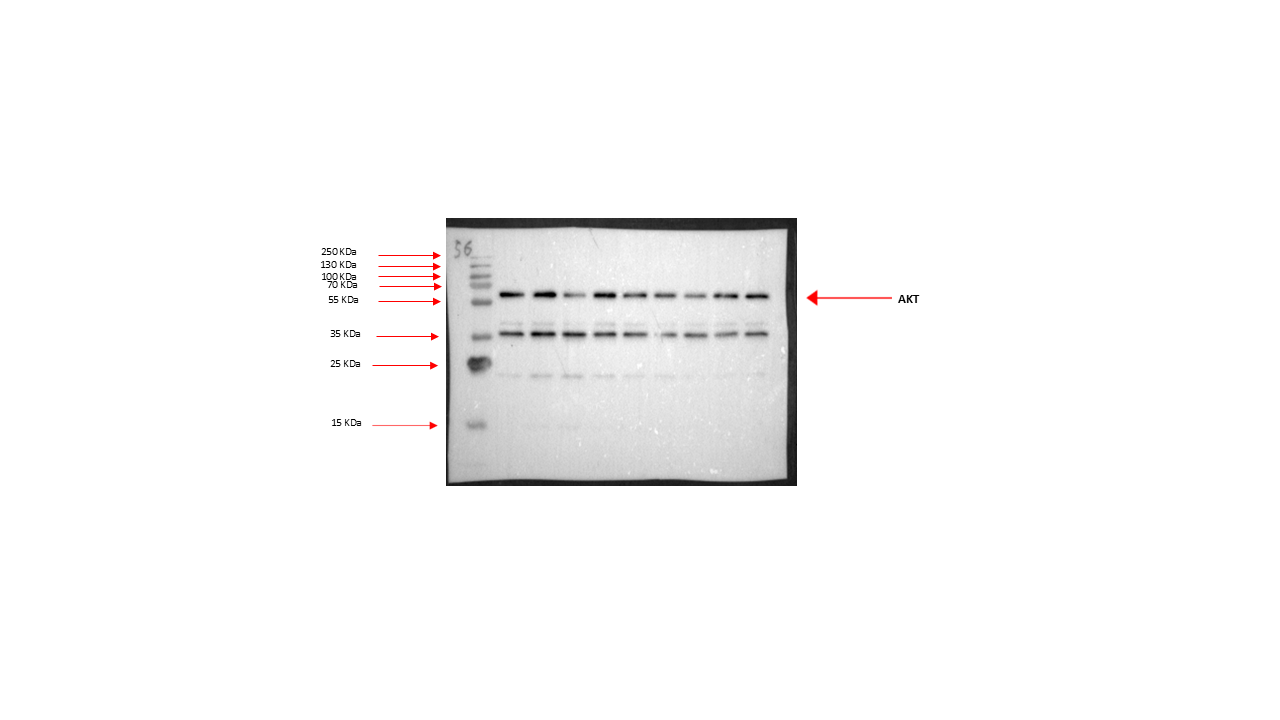

Supplement: Supplementary file 1 [file nutrients-16-04202-s001.zip › Figure S3.tif]

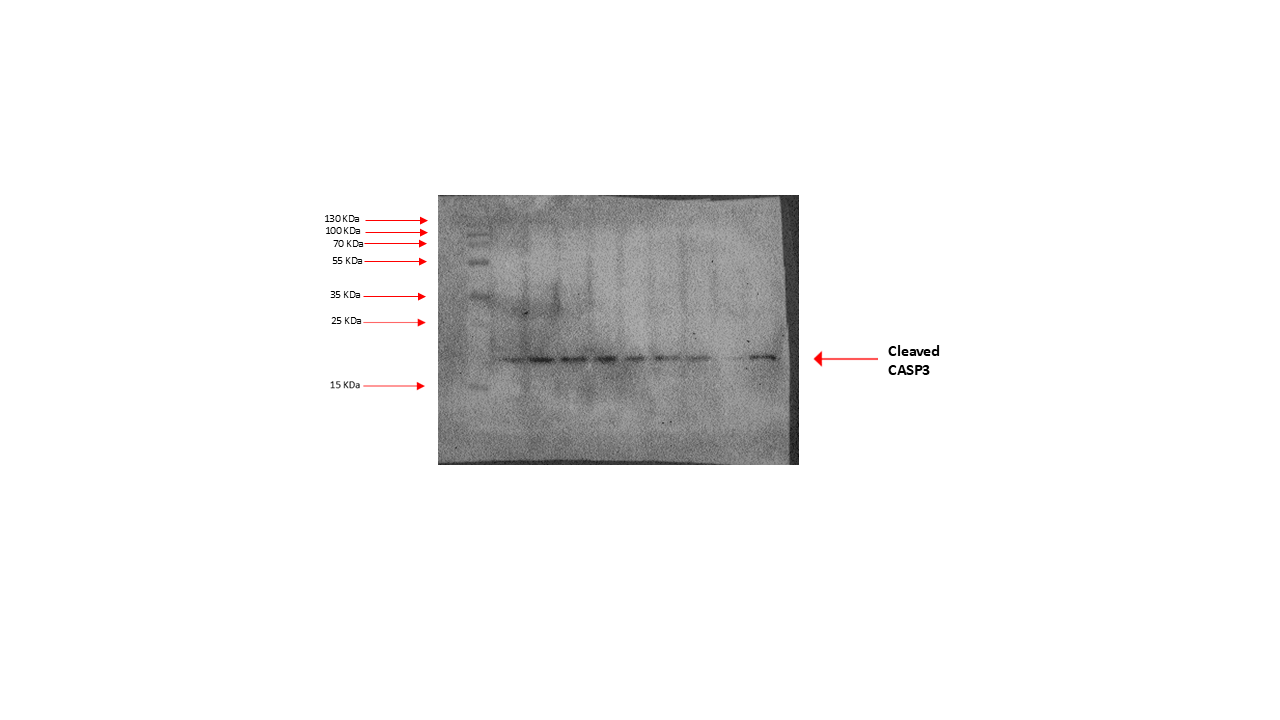

Supplement: Supplementary file 1 [file nutrients-16-04202-s001.zip › Figure S4.tif]

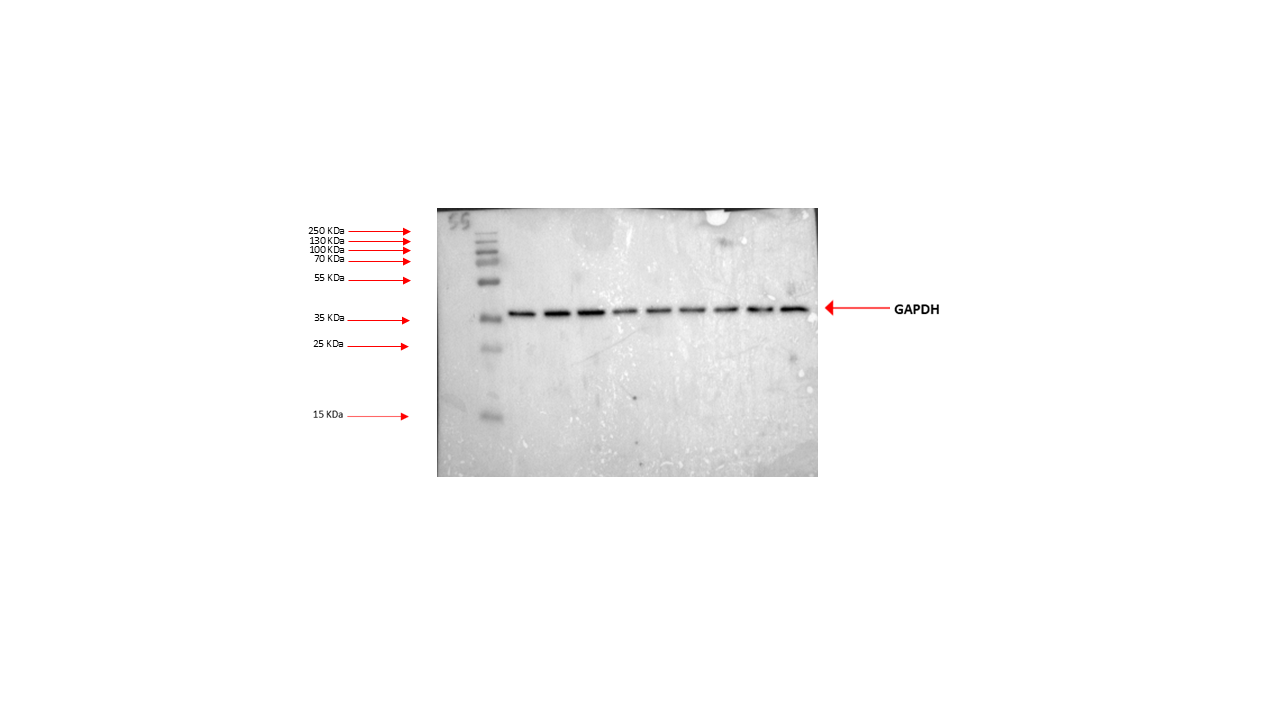

Supplement: Supplementary file 1 [file nutrients-16-04202-s001.zip › Figure S5.tif]
